# Evofosfamide sensitizes esophageal carcinomas to radiation without increasing normal tissue toxicity

**DOI:** 10.1016/j.radonc.2019.06.034

**Published:** 2019-12

**Authors:** Linda Spiegelberg, Stefan J. van Hoof, Rianne Biemans, Natasja G. Lieuwes, Damiënne Marcus, Raymon Niemans, Jan Theys, Ala Yaromina, Philippe Lambin, Frank Verhaegen, Ludwig J. Dubois

**Affiliations:** a Department of Precision Medicine, The M-Lab, GROW – School for Oncology and Developmental Biology, Maastricht Comprehensive Cancer Centre, Maastricht University, Maastricht, the Netherlands; b Department of Radiation Oncology (Maastro), GROW – School for Oncology and Developmental Biology, Maastricht Comprehensive Cancer Centre, Maastricht University Medical Centre, Maastricht, the Netherlands

**Keywords:** TH-302, Radiotherapy, Esophageal cancer, Short-term gut toxicity, Long-term lung fibrosis, Therapeutic index

## Abstract

**Background and purpose:**

Esophageal cancer incidence is increasing and is rarely curable. Hypoxic tumor areas cause resistance to conventional therapies, making them susceptible for treatment with hypoxia-activated prodrugs (HAPs). We investigated *in vivo* whether the HAP evofosfamide (TH-302) could increase the therapeutic ratio by sensitizing esophageal carcinomas to radiotherapy without increasing normal tissue toxicity.

**Materials and methods:**

To assess therapeutic efficacy, growth of xenografted esophageal squamous cell (OE21) or adeno (OE19) carcinomas was monitored after treatment with TH-302 (50 mg/kg, QD5) and irradiation (sham or 10 Gy). Short- and long-term toxicity was assessed in a gut mucosa and lung fibrosis irradiation model, sensitive to acute and late radiation injury respectively. Mice were injected with TH-302 (50 mg/kg, QD5) and the abdominal area (sham, 8 or 10 Gy) or the upper part of the right lung (sham, 20 Gy) was irradiated. Damage to normal tissues was assessed 84 hours later by histology and blood plasma citrulline levels (gut) and for up to 1 year by non-invasive micro CT imaging (lung).

**Results:**

The combination treatment of TH-302 with radiotherapy resulted in significant tumor growth delay in OE19 (*P* = 0.02) and OE21 (*P* = 0.03) carcinomas, compared to radiotherapy only. Irradiation resulted in a dose-dependent decrease of crypt survival (*P* < 0.001), mucosal surface area (*P* < 0.01) and citrulline levels (*P* < 0.001) in both tumor and non-tumor bearing animals. On the long-term, irradiation increased CT density in the lung, indicating fibrosis, over time. TH-302 did not influence the radiation-induced short-term and long-term toxicity, confirmed by histological evaluation.

**Conclusion:**

The combination of TH-302 and radiotherapy might be a promising approach to improve the therapeutic index for esophageal cancer patients.

## Highlights

• TH-302 combined with RT enhances tumor growth delay in esophageal cancer models.
• This combination does not increase normal tissue toxicity on the short and long term.
• The increased therapeutic index (1.38) paves the way for future clinical trials.

Esophageal cancer incidence in the Western world has risen over the past decades, with adenocarcinomas now being more prevalent than squamous cell carcinomas [1]. Alcohol intake, tobacco smoking and overweight are key risk factors, especially for adenocarcinomas. The disease is rarely curable with a 5-year overall survival of approximately 20% [2], [3]. Standard treatment consists of neoadjuvant chemoradiotherapy followed by surgery [4].

Like most solid tumors, esophageal cancers contain heterogeneously spread hypoxic regions [3], [5]. Tumor hypoxia negatively affects prognosis in multiple cancer types, including esophageal cancer [3], [6]. Hypoxia-activated prodrugs (HAPs) are designed to be selectively activated only under low oxygen tension, resulting in a hypoxia-specific release of their toxic effector. Evofosfamide (TH-302) is reduced to a radical anion by cellular reductases which, under normoxia, rapidly reacts with oxygen to generate the original prodrug. In the absence of oxygen, the radical anion is irreversibly fragmented releasing bromo-isophosphoramide mustard, which alkylates the DNA and is able to diffuse to surrounding cells creating a bystander effect [7]. Thus, in combination with conventional therapies, HAPs can be predicted to enhance treatment outcome.

Monotherapy efficacy has been established in different *in vivo* tumor models [8], [9], [10]. Improved therapeutic outcomes were found upon combination with chemotherapy, both preclinically [11], [12], [13], [14], [15] and clinically [16], [17], [18], [19]. TH-302 was well tolerated and evidence of anti-tumor activity was established. However, when supplemented to conventional chemotherapy [20], two phase III trials failed proving a benefit of TH-302. The combination of TH-302 with RT has not been as extensively studied. A few recent preclinical studies show that this combination effectively inhibited tumor growth [21], [22], [23], [24].

For a successful treatment, not only a reduction in tumor burden is crucial but also minimizing the risk of healthy tissues damage. These two factors are expressed as the therapeutic index. When combining novel therapies with standard regimes, extra care should be taken to assess normal tissue toxicity [25]. Adverse effects of RT include mucositis [26], a short-term effect greatly affecting quality of life and therapy continuity, and fibrosis, which manifests itself on the long-term and is frequently seen in lung tissue [27]. In the aforementioned preclinical studies combining HAPs with conventional therapies, normal tissue toxicity has not been evaluated.

In the present study, we therefore assessed *in vivo* whether TH-302 can sensitize esophageal carcinomas to RT and if this combination therapy results in increased short-term and/or long-term normal tissue toxicity using a gut mucosa and a lung fibrosis model, sensitive to acute and late radiation injury respectively.

## Materials and methods

### Cell lines and compound

Esophageal OE19 (adenocarcinoma) and OE21 (squamous cell carcinoma) cells were a kind gift from Dr. E. Hammond (CRUK/MRC Oxford Institute for Radiation Oncology, Department of Oncology, University of Oxford). H460 (non-small cell lung carcinoma) and OE21 cells were grown in RMPI-1640, while OE19 cells in DMEM all supplemented with 10% fetal bovine serum (Lonza). Cells were kept in a 5% CO_2_ incubator at 37°. For low oxygen concentrations an anoxic chamber (Don Whitley Scientific, UK) with <0.02% O_2_ was used.

TH-302, supplied by Threshold Pharmaceuticals, was dissolved in 0.9% NaCl to a concentration of 5 mg/ml for *in vivo* studies and in DMSO in different concentrations for *in vitro* studies.

### Cell viability assay

To determine if TH-302 increases intrinsic radiosensitivity, cells were seeded in 96-well microtiter plates and allowed to attach overnight. Cells were exposed to different oxygen conditions for 24 h until radiation. TH-302 was added (25 µM) 2 h before irradiation with a 225 kV X-ray tube (Philips) and washed out immediately after. TLD based dosimetry has been performed to calibrate the irradiator. Five days after irradiation, cell viability was determined by crystal violet staining and optical density (590-nm) was measured using a FLUOstar Omega plate reader (BMG Labtech). For each condition 6 technical replicates were used.

### Animal experiments

All animal experimental procedures were approved by the Animal Ethics Committee of Maastricht University (The Netherlands) and were in accordance with the EU Directive 2010/63/EU for animal experiments. For all experiments, female mice were monitored and weighed at least 3 times per week.

### Tumor growth delay

Esophageal cancer cells were resuspended in a mixture of serum-free medium and Matrigel (BD Biosciences) and injected (5 × 10^6^ cells) in the lateral flank of Nu-Foxn1-nu (NU/NU) mice. Tumor volume was determined by measuring the three orthogonal diameters using a Vernier caliper as previously described [22]. Upon an average volume of 256 ± 55 mm^3^ (OE19) or 232 ± 54 mm^3^ (OE21), mice (*n* = 8–10/group) were randomized between NaCl and TH-302 (50 mg/kg, i.p.) for five consecutive days (QD5). Two hours after the last injection, tumors were locally irradiated (sham or 8 Gy) using a linear accelerator (Varian, Palo Alto, CA) [28]. Tumor volume was monitored until 4 times the start volume (T4xSV) was reached. Animals for histology (*n* = 5/group) were sacrificed after the last TH-302 injection. Pimonidazole (60 mg/kg, i.p.; Bio-connect) and Hoechst 33342 (15 mg/kg, i.v.; Sigma–Aldrich) were administered 1 hour and 1 min before sacrifice, respectively. Tumors were excised, snap frozen and stored at −80 °C.

### Short-term normal tissue toxicity – gut

CD2F1 immune-competent mice were treated with NaCl or TH-302 (50 mg/kg, i.p., QD5). Two hours after the last injection, the abdominal area of the mice was irradiated (sham, 8 or 10 Gy) using the X-RAD225Cx (Precision X-Ray, North Branford, CT) [29] with two 40-mm square parallel-opposed fields under isoflurane anesthesia. Blood was drawn via cardiac puncture and gut tissue (proximal part of the jejunum) was collected 84 h after irradiation.

To assess gut toxicity in tumor-bearing mice, Nu-Foxn1-nu (NU/NU) immune-compromised mice were injected into the lateral flank with H460 (1 × 10^6^) or OE21 (5 × 10^6^) cells suspended in a mixture of serum-free medium and Matrigel (1:1). Upon reaching an average tumor volume of 257 ± 59 mm^3^ (H460) or 221 ± 22 mm^3^ (OE21), animals were randomized across the treatment schedule as described above.

### Long-term normal tissue toxicity – lung

C57BL/6 immune-competent mice received injections of NaCl or TH-302 (50 mg/kg, i.p., QD5). Two hours after the last injection, approximately 20% of the right lung was sham-irradiated or with 20 Gy using the X-RAD 225Cx with 5-mm square parallel opposed fields as previously described [30] (Fig. 4A). Delineation of the left and right lung on all time-points was performed manually. At 3, 6, 9, 10, 11 and 12 months post-RT, thorax CT images (80 kVp, 2.5 mA, 100 µm resolution) were acquired to assess changes in electron density. Deformable image registration and image analyses were performed as has been described previously, with the exception that non-rigid image deformations were performed using the Elasix toolbox [31], [32].

### Radiation planning and delivery

For precision irradiations, the preclinical research platform X-RAD 225Cx (Precision X-Ray, North Branford, CT) was used according to the ACROP guidelines [33] (see supplementary information).

### Ex vivo analyses

See Supplementary information.

### Statistics

GraphPad Prism software (version 5.01 for Windows) was used to perform statistical analyses. One-way ANOVA with Tukey’s multiple comparison tests were used to determine the statistical significance of differences between independent groups of variables. For all tests, a *P* < 0.05 was considered significant.

## Results

### Therapeutic efficacy of TH-302 in combination with radiotherapy

**Fig. 1:**
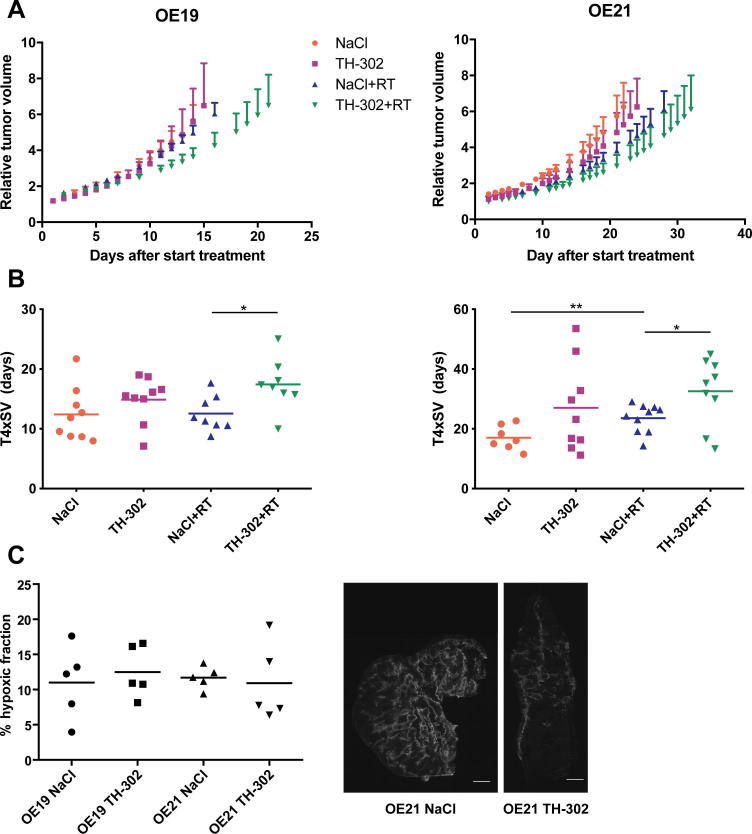
Effect of TH-302 in combination with RT on tumor growth and hypoxia. Mice with esophageal adenocarcinoma (OE19, *n* ≥ 8) or squamous cell carcinoma (OE21, *n* ≥ 7) xenografts were (sham-) treated with TH-302 and/or RT (8 Gy) and tumor volume was measured three times a week. Tumor growth curves (A), time to reach 4 times start volume (T4xSV) (B) and hypoxic fraction (C) are shown for both tumor models. Examples of hypoxic staining in tumors for NaCl and TH-302 treated mice are shown in (D) in which white areas are hypoxic. Data, mean ± SEM. *P < 0.05, **P < 0.01.

The effect of the combination of TH-302 and RT was assessed in two esophageal cancer xenograft mouse models. In both models, TH-302 monotherapy showed inhibition of tumor growth, albeit not significantly, while combination with 8 Gy single dose irradiation significantly reduced tumor growth (Fig. 1A). Time to reach four times start volume (T4xSV, Fig. 1B) increased by TH-302 monotherapy (12.4 ± 4.5  days to 14.5 ± 3.8 and 17.1 ± 4.0 to 27.0 ± 14.9 days for OE19 and OE21 respectively). Radiation (8 Gy) did not affect T4xSV for the radioresistant OE19 (12.6 ± 3.0 days), while in OE21 T4xSV was significantly increased to 23.5 ± 4.7 days compared to sham treatment (*P* = 0.01). The combination of RT with TH-302 resulted in the largest tumor growth delay in both models. Compared to RT alone, the combination therapy significantly increased T4xSV from 12.6 ± 3.0 to 17.4 ± 4.2 days for OE19 (*P* = 0.02) and from 23.6 ± 4.7 to 32.6 ± 11.1 days for OE21 (*P* = 0.03), resulting in enhancement ratios of 1.38 and 1.39 respectively.

To evaluate the effect of TH-302 on the levels of hypoxia, the hypoxic fraction was assessed immediately after TH-302 treatment in tumor-bearing animals from a parallel histology study (Fig. 1C and D). For OE19 there was no difference between sham or TH-302 treated animals (10.9 ± 5.2% vs 12.5 ± 3.7%). Although for OE21 tumors there was also no significant difference between sham and TH-302 treatment (11.7 ± 1.6% vs 10.9 ± 5.5%), 3 out of 5 tumors treated with TH-302 did have a lower hypoxic fraction.

**Supplementary Fig. 1:**
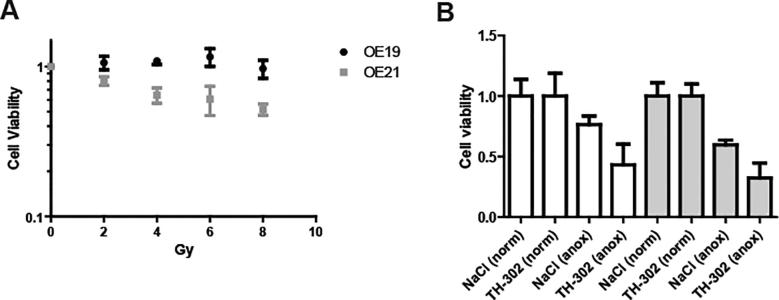
Crystal violet cell viability assay shows greater radiosensitivity of OE21 than OE19 cells under normoxia (A). TH-302 only affects cell viability for both OE19 (white bars) and OE21 (gray bars) under anoxic conditions (B). Data show mean ± SEM of 3 independent experiments.

The lower OE19 radiosensitivity was confirmed in an *in vitro* cell viability assay (Suppl Fig. 1A). This assay also showed that TH-302 was only activated under anoxic conditions and that the combination of RT and TH-302 resulted in the lowest cell viability (Suppl Fig. 1B), consistent with the *in vivo* data.

### Short-term normal tissue toxicity

**Fig. 2:**
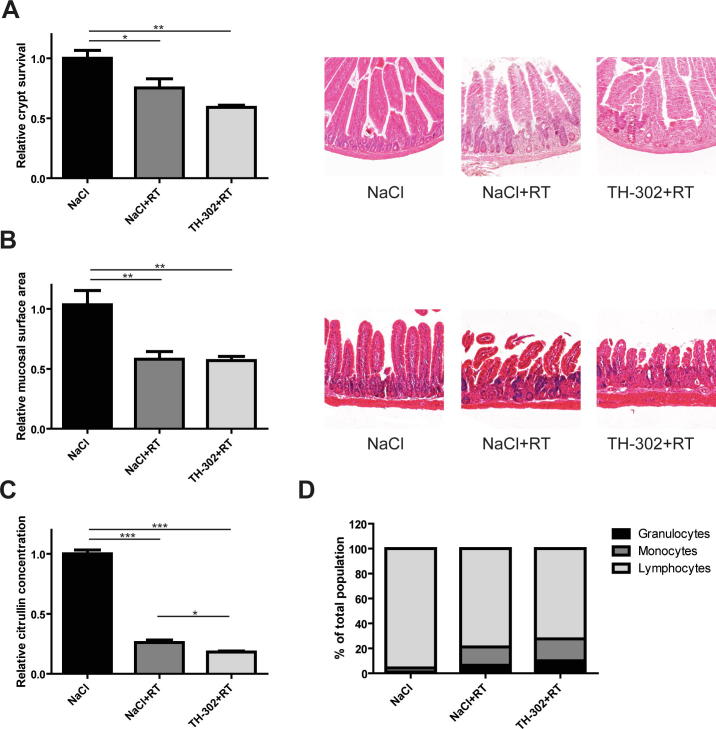
Effect of TH-302 on short term radiation-induced toxicity. Immune-competent mice were treated with TH-302 and irradiated with 8 Gy in the abdominal area. 3.5 days later, toxicity in the gut and blood was assessed. Relative crypt survival and mucosal surface area in the jejunum with representative images are shown in (A) and (B). Relative plasma citrulline levels and proportion of granulocytes, monocytes and lymphocytes in whole blood are shown in (C) and (D), respectively. Data (saline *n* = 3, other groups *n* ≥ 5), mean ± SEM. *P < 0.05, **P < 0.01.

**Supplementary Fig. 2:**
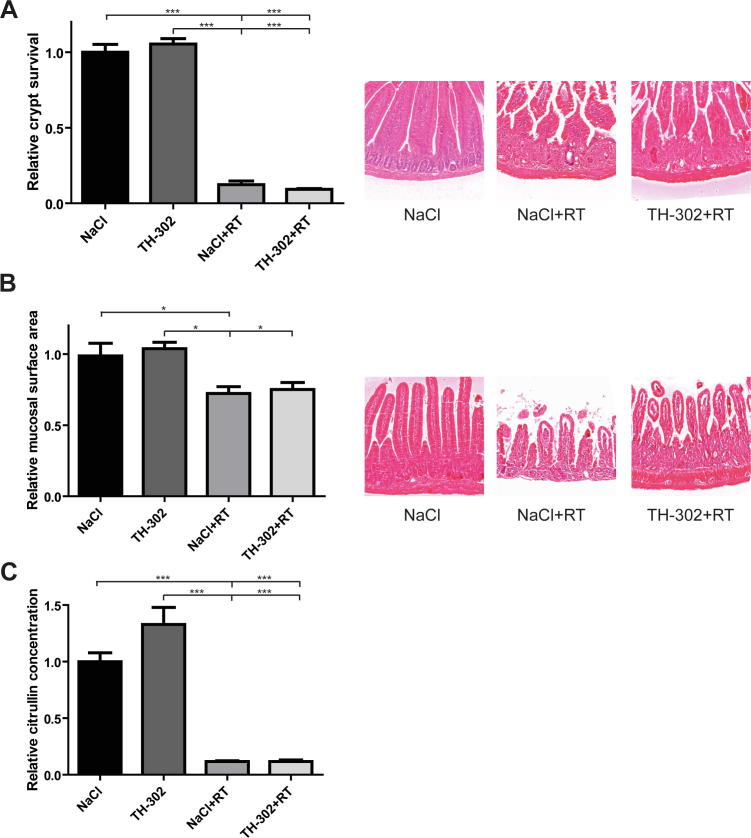
The effect of the combination of TH-302 and radiotherapy on acute radiation toxicity in the jejunum of immunocompetent mice. Mice were treated with control (NaCl) or TH-302 (50 mg/kg) for 5 consecutive days followed by radiotherapy (sham or 10 Gy). 3.5 days after radiotherapy, mice were sacrificed and crypt survival (A) and mucosal surface area (B) and plasma citrulline concentration (indicative of gut damage, C) were assessed. Data (*n* ≥ 5, except MSA TH-302 group n = 4) show mean ± SEM.

The effect of RT and the possible additional effect of TH-302 on short-term normal tissue toxicity were assessed using a gut toxicity model. This standardized model has been widely used to screen for acute radiation toxicity [34] and was chosen here as a representative model for GI tract mucosa. Crypt survival (Fig. 2A; 25%, *P* < 0.05) and mucosal surface area (Fig. 2B; 42%, *P* < 0.01) decreased upon RT. Plasma citrulline levels, indicative for radiation damage to the gut, also declined (*P* < 0.001) after 8 Gy (Fig. 2C; 74% decrease compared to controls). The combination of TH-302 and RT resulted in a small reduction in citrulline concentration compared to RT alone (*P* < 0.05), whereas there was no additional effect of TH-302 on crypt survival and mucosal surface area. TH-302 monotherapy did not affect short-term toxicity and the combination with 10 Gy irradiation showed a comparable pattern (Suppl Fig. 2A–C). FACS analysis to evaluate the effect on immune cell subpopulations present in the blood showed that the proportion of lymphocytes in the blood decreased by 17% (96% in sham vs 79% in RT treated mice), while the proportion of granulocytes and monocytes increased (by 5% and 12% respectively; Fig. 2D). The combination therapy did not alter these percentages.

**Fig. 3:**
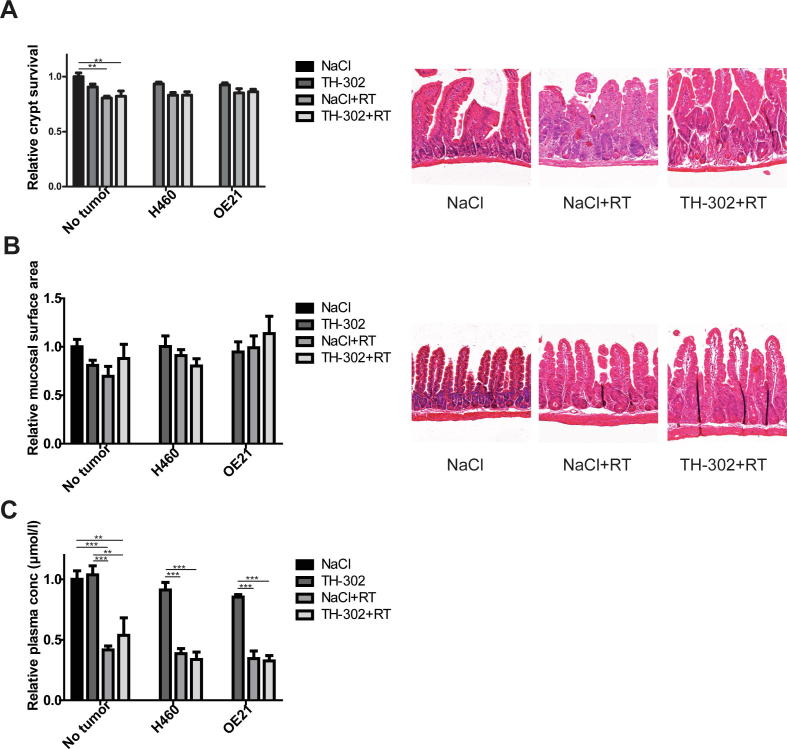
Effect of TH-302 on short term radiation-induced gut toxicity in tumor bearing mice. Immune-compromised mice without (*n* = 6, except MSA TH-302 group *n* = 4) tumor or bearing H460 (*n* ≥ 6, except crypt TH-302 group *n* = 5) or OE21 (*n* = 6) tumors were treated with TH-302 and irradiated with 8 Gy in the abdominal area. 3.5 days later, toxicity in the gut and blood was assessed. Relative crypt survival and mucosal surface area with representative images of the jejunum are shown in (A) and (B). Relative plasma citrulline levels are shown in (C). Data, mean ± SEM. *P < 0.05, **P < 0.01.

To mimic the clinical situation more closely, the gut toxicity study was also performed in tumor-bearing animals to assess whether potential leakage of the active metabolite from the tumor influenced gut toxicity. The H460 tumor model was chosen because it was previously shown that it is highly responsive to TH-302 treatment [22] and the OE21 model because it was more radiosensitive than OE19. Crypt survival (Fig. 3A) and mucosal surface area (Fig. 3B) decreased after RT, although not as explicit as in non-tumor bearing, immune-competent mice. Citrulline levels (Fig. 3C) declined after RT in non-tumor bearing immune-compromised animals (58% decrease), H460 tumor-bearing animals (61%) and OE21 tumor-bearing animals (66%). For all parameters and all three groups, the combination of TH-302 and RT did not increase intestinal damage compared to radiotherapy alone.

**Supplementary Fig. 3:**
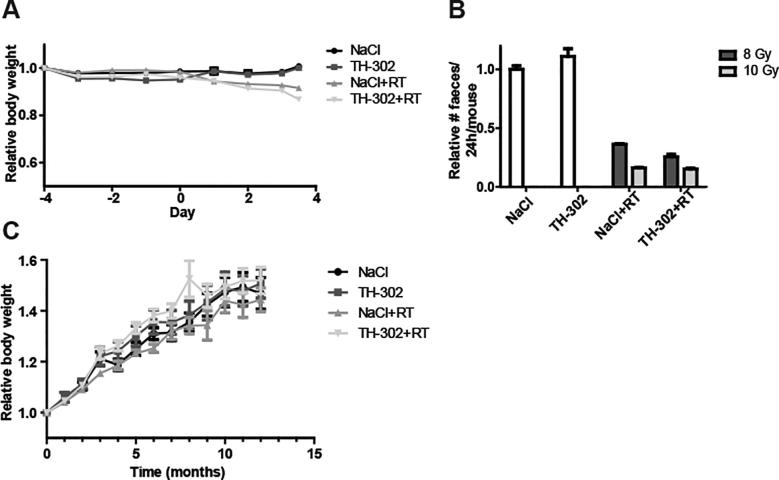
Relative body weight of mice in short-term toxicity experiments. Day-4 = start treatment (TH-302 50 mg/kg, QD5) or saline, day 0 = radiotherapy (A). Relative number of feces per mouse after 8 or 10 Gy (B). Relative body weight of mice in long-term toxicity experiment after radiotherapy (C, RT at *t* = 0).

Besides gut toxicity, general health was evaluated. No clinical symptoms of distress were observed. Body weight (Suppl Fig. 3A) decreased minimally in all RT treated animals, with a slightly larger decrease in the combination therapy groups. The effect of RT was more discernibly reflected in the amount of feces (Suppl Fig. 3B) that dropped to less than half to that of non-irradiated animals.

### Long-term normal tissue toxicity (lung)

**Fig. 4:**
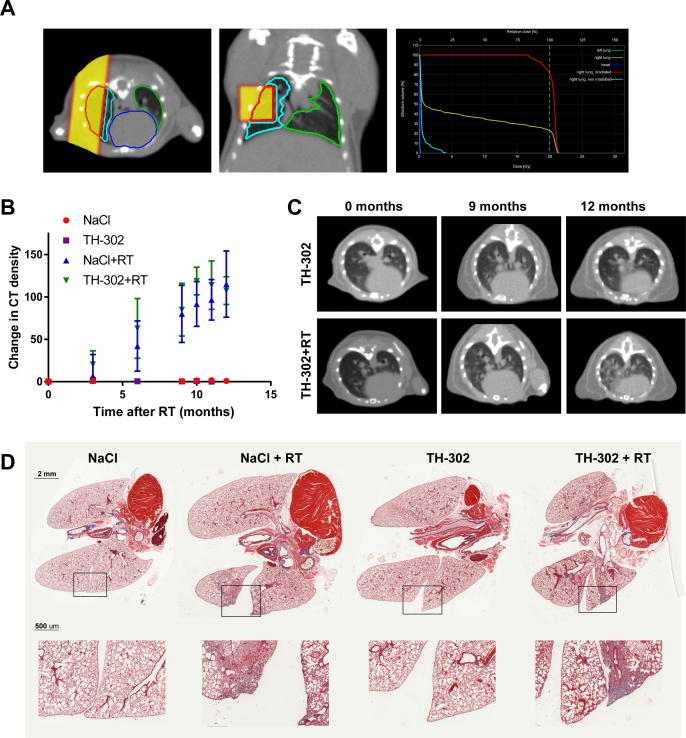
Effect of TH-302 on long term radiation-induced fibrosis in the lungs. Immune-competent mice were (sham-) treated with TH-302 and approximately 20% of the right lung was (sham-) irradiated with 20 Gy. Lungs were examined for fibrosis on CT scans for up to 1 year after irradiation. Example of a lung irradiation treatment plan, showing the beam dose deposition in yellow on axial (left) and coronal (middle) slides is shown in (A). Dose–volume histograms with the different tissue structures are shown on the right. Changes in CT density (irradiated part versus unirradiated part of the right lung) representative for fibrosis are shown in (B) (sham-irradiation *n* = 6, irradiation *n* = 8). (C) Representative axial CT slides for a mouse treated with TH-302 (upper) and TH-302 with radiotherapy (lower). Representative Masson’s Trichrome stained lungs are shown for each treatment arm in (D). Upper row shows full lungs/heart section (2 mm scale bar), while lower row shows magnification (500 μm scale bar) of the indicated area.

For the long-term effects of radiotherapy in combination with TH-302, a lung fibrosis model was used. Lungs were partially irradiated with 20 Gy (Fig. 4A), as this was shown before to result in clear changes in CT density representing fibrosis [30], [31]. Body weight steadily increased during the duration of the study (Suppl Fig. 3C). CT density increased over time in the irradiated part of the right lung (Fig. 4B and C, *P* < 0.001). The addition of TH-302 to radiotherapy did not alter changes in CT values. Sham-irradiated control animals, treated with NaCl or TH-302, did not show an increase in CT values. Histology confirmed fibrosis in the irradiated part of the right lung as seen by the loss of alveolar structure and collagen deposition (Fig. 4D). TH-302 addition did not alter histological appearance.

## Discussion

In this study we showed that the combination of evofosfamide (TH-302) and RT in two esophageal cancer xenograft models results in an enhanced tumor growth delay. Our data are in line with recently published studies with different tumor models [21], [22], [23]. In addition, we showed little or no additional toxicity when supplementing TH-302 to RT on the gut (short-term) and lung (long-term). Together, this resulted in an increased therapeutic index.

Although the OE19 model was more resistant to therapy (to RT, TH-302 monotherapy and the combination) than OE21, both models showed an increased time to reach four times start volume (T4xSV) upon TH-302 combination with RT *in vivo*. TH-302 monotherapy affected T4xSV in the both models with a more pronounced difference in OE21 tumors. This is in line with the effect of TH-302 on the hypoxic fraction, which was not influenced in the OE19 model, but decreased for 3 out of 5 tumors in the OE21 model. Similar results have been observed for the alkylating-like agent cisplatin, due to induction of autophagy [35], which could explain the observed difference in response to TH-302 in our study. It once more stresses the fact that each cancer type behaves differently and personalized medicine strategies are of utmost importance [36].

In this study short-term normal tissue toxicity was evaluated in both immune-competent and immune-compromised mice. Tumor-bearing, immune-compromised mice were taken along because in this model TH-302 is activated in hypoxic tumor cells and we could confirm that the active metabolite was not able to produce additional damage in the gut. In immune-competent non-tumor bearing mice, RT had a larger effect, in agreement with previous studies [37], [38]. The effect of total body irradiation on mucosal surface area was more pronounced in C57Bl/6 immunocompetent (13% decrease, [38]) compared to NMRI-nu/nu immunocompromised (7% decrease, [37]) animals. This could be explained by the fact that radiation-induced acute damage to the gut is, besides the direct effect of RT, also due to inflammation for which an intact immune system is required [39], aggravating the observed effects on mucosal surface area in immunocompetent animals. Surprisingly, while in immune-compromised mice TH-302 had no additional effect on radiation-induced damage to the gut, in immune-competent mice there was a small difference in the level of citrulline indicating more damage in the combination therapy treated group. This could be related to changes in the distribution of white blood cells. RT alone lowered the proportion of peripheral lymphocytes, which is a well-known effect [40], [41], and the combination therapy further marginally, although not significantly, lowered this proportion. This could be due to the activation of TH-302 in the relatively hypoxic bone marrow where lymphocytes are produced [42]. However, the biological implications of the minor differences in gut toxicity are expected to be very low and not influence outcome.

TH-302 did not increase radiation-induced toxicity on the long term, as assessed by changes in CT density of partially irradiated lungs representing fibrosis. It was described before that partial irradiation allows higher doses to be used compared to large field irradiation and that there is a clear dose–volume effect [30], [31]. Our results in irradiated and non-irradiated animals are in agreement with these studies, indicating the reproducibility of this method. We followed up animals until 12 months post-RT (compared to 9 months in the aforementioned studies) and it was shown that after 9 months, CT density did not significantly increase anymore, confirming the usefulness of this time-point for further studies. Furthermore, histological evaluation for fibrosis confirmed the changes in CT density upon irradiation, in agreement with previous studies [30]. TH-302 did not affect histological appearance.

The therapeutic index of a treatment takes into account the therapeutic benefit and associated normal tissue toxicity. Nonetheless, preclinical studies on TH-302 have not in depth studied its effect on normal tissues, especially not for the combination with RT. Here we show a favorable tumor growth inhibition by the combination of TH-302 and RT over RT alone in two esophageal cancer models, expressed as an enhancement ratio (ER) of 1.38 (average of the two models). The overall ER of the different tissue toxicity parameters tested is 0.995 resulting in a therapeutic index of 1.38, indicating a clear benefit of the combination therapy.

Thus far TH-302 has been primarily tested in combination with chemotherapy, preclinically as well as clinically. After positive phase I and II studies, it was reported in two phase III trials that TH-302 in combination with standard chemotherapy (gemcitabine or doxorubicin) did not improve overall survival in patients with advanced pancreatic cancer (MAESTRO; NCT01746979) or soft tissue sarcoma [20]. However, significance was nearly reached in the MAESTRO trial. Within these studies, patients were however not stratified by tumor hypoxic status, potentially explaining the observed lack of clinical benefit. Hence, the hypoxic fraction of tumors varies widely, even within the same tumor type [43], [44] and the effectiveness of TH-302 greatly depends on hypoxia [22]. Therefore, without patient stratification, the effect of the combination therapy is likely to be underestimated. Furthermore, it was recently stated that the effectiveness of TH-302 may be partly antagonized by doxorubicin due to electron transfer from TH-302 to doxorubicin [45]. In this way, the formation of the effector of TH-302 (bromo-isophosphoramide mustard) is blocked, which could account for the lack of effect of the combination therapy in the soft tissue sarcoma trial. Although the path of TH-302 in clinical studies has stalled after these phase III trials, a new clinical trial regarding the combination with immunotherapy is currently recruiting patients (NCT030098160).

The combination of TH-302 with RT is not as thoroughly investigated. In particular, studies should investigate the combination effect in clinically relevant settings, focusing on fractionation schedules. So far, only one study investigated the efficacy of TH-302 combined with fractionated radiotherapy (3 × 2 Gy) and observed effects on therapeutic outcome dependent on treatment schedule and tumor type, most probably related to the status of HAP-activating oxidoreductases [31]. Clinical trials have not been carried out yet, although preclinical evidence, including this study, is showing that this might be a promising combination therapy. Additionally, we show here that normal tissue toxicity is not increased by the combination of TH-302 and RT. This combination may be favorable over chemotherapy due to lesser systemic normal tissue toxicity. Much effort has been done to maximally spare normal tissues using different modes of RT, such as intensity modulated radiotherapy (IMRT) or proton therapy [46], [47]. Furthermore, toxicity to normal tissues due to RT is localized to the tumor site, while chemotherapeutic toxicity is systemic and can affect multiple organs. The maximal tolerated dose (MTD) of TH-302 when combined with chemotherapy is much lower than the MTD of TH-302 monotherapy [18], [19]. Possibly, higher dosing schedules can be used when combining TH-302 with RT leading to improved therapeutic benefit. The clinical trial protocol to test this hypothesis has been recently published [48].

In conclusion, we show that the combination of TH-302 and RT influences therapeutic efficacy to a different extent in two esophageal cancer models, with only minor effect on radiation-induced normal tissue toxicity. To pave the way for clinical testing of this combination, studies in various animal models should focus first on different clinically relevant treatment schedules. With regard to potential clinical trials, it is strongly recommended to carefully consider trial design and incorporate hypoxia stratification to overcome interpretation difficulties that have arisen in the aforementioned trials.

## Declaration of Competing Interest

None.
