# Supplementary material for: Evofosfamide sensitizes esophageal carcinomas to radiation without increasing normal tissue toxicity
Source: Radiother Oncol. 2019 Dec;141:247–55. doi: 10.1016/j.radonc.2019.06.034 (PMC6913516; doi:10.1016/j.radonc.2019.06.034)
Supplement: Supplementary data 1 [file mmc1.docx]

**Radiation planning and delivery**

Absolute dosimetry was performed following the AAPM TG-61 protocol using the in-air method [1]. Irradiations were performed at 225 kVp, 13 mA with an inherit 0.8 mm beryllium filter and an additional 0.3 mm copper filter, resulting in a spectrum with a half value layer of 0.98 mm copper. The 40-mm square beam delivered a dose rate of 3.6 Gy/minute at the source-to-isocenter distance of 303.6 mm. The 5-mm square beams had a full width half maximum of 4.9 mm and a penumbra size of 0.5 mm (20% - 80% of maximum dose) at the isocenter and a dose rate of approximately 2.5 Gy/minute at the dose prescription point. All calculated dose values were scored as dose-to-medium, transport-in-medium. All animals were treated in feet first prone position.

Radiation planning was performed using SmART-Plan version 1.5.0 [2]. CT acquisition parameters, CT number to density calibration and medium segmentation, were performed as reported previously [3]. SmART-Plan was calibrated to our X-RAD 225Cx, all collimators were validated using radiochromic film measurements.

**Ex vivo analyses**

*Plasma citrulline*

Plasma citrulline measurement were determined using an electro-spray ionization ultra-performance liquid chromatography tandem mass spectrometry (UPLC-MS/MS) [4].

*Flow cytometry*

The number of immune cell subpopulations present in the blood was analyzed using FACSCanto II flow cytometry (FACS, BD Bioscience). Serum was separated from whole blood, erythrocytes were lysed (RBC lysis buffer, e-Bioscience, 00-4300-54). The remaining immune cells were suspended in FACS buffer (PBS + 1% FCS) and incubated with Fc-block (BD BioSciences, 553142) to avoid nonspecific binding. Staining was performed using CD3-FITC and CD45-V500 (BD BioSciences, 555274 and 561487). The total CD45+ immune cells were selected from the viable population of cells (filtered for debris and doublets) and further classification was made into lymphocytes, granulocytes and monocytes based on CD3 positivity and size/forward/side scatter.

*Histology*

Crypt survival

Transverse 5 µm sections of paraffin-embedded jejunum were cut and stained with hematoxylin and eosin (H&E). Surviving crypts were counted in 2-4 sections 0.5 mm apart from each other. Crypt survival was expressed as average number of crypts per circumference.

Mucosal surface area

Longitudinal sections of the jejunum were cut in 5 µm sections. For each mouse, 2 pictures at 10x magnification were taken. Using ImageJ software (National Institute of Health), mucosal surface area was measured as follows: a region of interest was manually drawn that included the villi, but excluded basal membrane and crypt area. An automated threshold above background was applied and the area was extracted. The length of the accompanying basal membrane was measured and the mucosal surface area was thus expressed as the mucosal surface area/length of basal membrane.

Lung fibrosis

Five µm thick sections were deparaffinized and stained for hematoxylin (Klinipath) and eosin (VWR) or for collagen using a Masson’s trichrome stain (Sigma-Aldrich) as previously described [5].

Hypoxic fraction

Assessment of the hypoxic fraction of tumors was done on frozen tumors as previously described [6]. Briefly, tumors were sectioned and stained for hypoxia (pimonidazole), using the primary antibody rabbit anti-pimonidazole (1:150; HP3-1000, Bio-connect) and secondary goat anti-rabbit Alexa594 (1:500, Invitrogen). Stained whole tumor cross-sections were scanned for pimonidazole using ImageJ, viable tumor tissue was delineated and the relative hypoxic fraction within the viable tumor area was calculated as previously described [7].

[1] Ma CM, Coffey CW, DeWerd LA, et al. AAPM protocol for 40-300 kV x-ray beam dosimetry in radiotherapy and radiobiology. Medical physics 2001;28:868-93.

[2] van Hoof SJ, Granton PV, Verhaegen F. Development and validation of a treatment planning system for small animal radiotherapy: SmART-Plan. Radiother Oncol 2013;109:361-6.

[3] Granton PV, Dubois L, van Elmpt W, et al. A longitudinal evaluation of partial lung irradiation in mice by using a dedicated image-guided small animal irradiator. International journal of radiation oncology, biology, physics 2014;90:696-704.

[4] Waterval WA, Scheijen JL, Ortmans-Ploemen MM, Habets-van der Poel CD, Bierau J. Quantitative UPLC-MS/MS analysis of underivatised amino acids in body fluids is a reliable tool for the diagnosis and follow-up of patients with inborn errors of metabolism. Clinica chimica acta; international journal of clinical chemistry 2009;407:36-42.

[5] De Ruysscher D, Granton PV, Lieuwes NG, van Hoof S, Wollin L, Weynand B, et al. Nintedanib reduces radiation-induced microscopic lung fibrosis but this cannot be monitored by CT imaging: A preclinical study with a high precision image-guided irradiator. Radiother Oncol. 2017;124:482-7.

[6] Peeters SG, Zegers CM, Biemans R, et al. TH-302 in combination with radiotherapy enhances the therapeutic outcome and is associated with pretreatment [18F]HX4 hypoxia PET imaging. Clinical cancer research : an official journal of the American Association for Cancer Research 2015.

[7] Yaromina A, Kroeber T, Meinzer A, et al. Exploratory study of the prognostic value of microenvironmental parameters during fractionated irradiation in human squamous cell carcinoma xenografts. International journal of radiation oncology, biology, physics 2011;80:1205-13.
